# Supplementary material for: Acceptance and compliance with micronutrient powder (MNP) among children aged 6–23 months in northern Nigeria
Source: PLOS Glob Public Health. 2022 Oct 17;2(10):e0000961. doi: 10.1371/journal.pgph.0000961 (PMC10022258; doi:10.1371/journal.pgph.0000961)
Supplement: S1 Dataset — (ZIP) [file pgph.0000961.s008.zip › S8_Dataset/In-depth Interviews.zip!/In-depth Interviews/060715_K_J_TA_2_CG.pdf]

A PHASE TWO (2) HOME-FEEDING TRIAL IN-DEPTH INTERVIEW WITH A MALE CAREGIVER AT RUGA COMMUNITY, JEGA LOCAL GOVERNMENT AREA OF KEBBI STATE

ON 6TH JULY, 2015

CONDUCTED AND TRANSCRIBED BY TIMOTHY AUTA

I: Please describe for me the people in your immediate family living in your household.

R: Ok. As regard to what?

I: How many you are in the family, number of children and gender of family composition.

R: The children should be up to seven. Alhamdulillah.

I: How many wives?

R: One wife.

I: What about the gender of the children?

R: I have four female and three male children

I: Thank you. We have heard of food challenges for young children in this community related to the seasons. What mechanisms could be employed in coping these food challenges?

R: This issue of seasonal foods is easy and is difficult. At the time when the farmers are assisted, we have enough food that sustains us all through the seasons. Sometimes, the rainy season would have gone far before we get fertilizer and other things we need for cultivation. Even millet sometimes doesn’t do well because of that. It is when they are about flowering that they come with fertilizer. I feel it can be addressed if fertilizer is provided on time.

I: Besides this, what other mechanisms could be used?

R: If we can get assistance from the government as I said earlier, we will have enough food as expected.

I: Thank you for the suggestion. Please tell me how food challenges affect infant and young child feeding.

R: Honestly, it affects them because you will see an infant with a mother who doesn't have good food to eat; there will be problem with the mother's breast milk. If a child has started feeding on supplementary food, we do give them cow milk. But when we don't have foods for our cattle, we move them to the Southern part of the country for grazing. You see, like our children, if they don't get that milk, it does affect them since we don't go to town to buy anything that will help them. But if we can be getting something to augment the breast milk they are taking, it will help them a lot.

I: Thanks for this explanation. One thing we heard in other interviews that caregivers often give their children 6-23 months "tea". Could you define or explain "tea" to me?

R: Honestly, we here don't use it, only in the cities.

I: Now, I want us to talk about the MNP that you received. Could you tell me about your experience with the product?

R: When it was brought to my wife, I was not at home I was in the Southern part of the country with the cattle. But when I came back, my wife told me and showed me the MNP that was brought by your people and she said it is to be use for a period of time; one per day, for sixty days, that's two months. It is very beneficial and I am grateful to the government. May they live long and God perfect everything.

I: OK. What are the positive aspects of your experience with the MNP?

R: Well, what will make me to see it... some problems like tamuwa, we do see it in other areas but God has not allowed it to happen around here. We have seen how children develop tamuwa and the health consequences and we now see people who want help us and we are following their instructions well, God helping it doesn't occur to ones child. I see it as deliverance and of trust. Why? Because of its protection, big one. When you see a problem in others places and it doesn't come to you and you're still getting assistance, you just thank God.

I: OK. Thank you so much for that information. Tell me about your perceived benefits of this MNP to the child?

R: Honestly, you know things must be studied very well. I may not be able to tell you how it will benefit him but based on the problems we have seen in other places and it has not come to us, it makes me know it is beneficial.

I: On the child you're giving this MNP, what benefit or good changes have you observed?

R: Like in health of the child?

I: Yes.

R: Honestly, I will say we have seen benefits. Because, the fever he often used to experience has drastically reduce. You will always see him playing around always.

I: Thank you for that information. Could you please tell me about any general challenges you had during the MNP trial?

R: Challenges.... honestly, I will say I didn't see any challenge in using it appropriately.

I: Ok. Could you tell me some specific barriers that made it hard to use the MNP appropriately?

R: I cannot categorically say there are barriers or not. This was brought in my absence and i don't know whether there was any barrier experienced in my absence. But to the best of my knowledge, there is known.

I: Uh hmm.

R: Yes.

I: Ok. Some caregivers mentioned that it was hard to store the MNP without it being taken by others or eaten by rodents. What was your experience?

R: That one I will say it is carelessness on the part of the caregivers. If someone says he/she kept it and others took it, it means he/she didn't keep it at the right place. If one keep it at the right place, he will be the only one that can have access to it. On the issue of rodents, there is a place one can put it where rodents cannot take it, if one really cares about it. You see, anytime they need to give the child, they go and pick it. But where caregiver will store it,

after giving it to the index child in the presence of other children, the other children will definitely want to have a feel of it. It won't be good.

I: What were the perceived side effects of the MNP on the child?

R: No. I have not seen any side effect.

I: Silence.... Some mothers indicated that it might be hard to remember to use MNP. What was your own experience?

R: Well. For that, if it is said that female caregivers don't remember to give the MNP, did the male caregiver remind her? The father supposed to have asked whether the mother has given it to the child and if she says no, you ask her to give immediately. You have helped her to remember. The mother can easily forget because of the domestic activities she is engaged in. But if the she is reminded, she won't forget totally.

I: OK. What solutions could you suggest that to address each of the above mentioned barriers or challenges?

R: Honestly, the ways most suitable is for the father to also take care of the family. If the mother is left alone with all these things, she may not remember always. If the father is assisting by telling her the time to use, she will always remember. For the storage of the MNP, there should be a safe place to always keep it. This will help in ensuring that the product is used to the last day.

I: OK. Thank you for the suggestions. Now, let's talk about the child's feeding and response to the MNP. Tell me how you feed the MNP to the child usually.

R: Briefly, just as she told me how to give it, it is one sachet one gives to the child per day.

I: Silence....

R: Since I was not at home when they brought it, my question is that if one starts using it in the morning the first day, is it s must for one to continue using in the morning every day? That is the question I couldn't ask.

I: OK. No problem, you will have the opportunity to ask questions later. What foods do you mix the MNP with?

R: The foods we mix it with are cow milk and koko.

I: What time do you give the child the MNP?

R: We mostly give the child in the morning. When the cattles are here, after collecting milk from the cow, i bring it and mix a little with the MNP to give the child.

I: OK. To what extent does the child likes or dislikes the taste of the MNP?

R: Honestly, since .... some children when they see that they are preparing the food, they will be looking...

I: (Interrupted) I want to hear about your child.

R: Alhamdulillah. When he sees that they are preparing food for him, he will be looking at the mother. Sometimes, you see him trying to grab it with his hand.

I: What about the taste?

R: He likes it.

I: OK. How has the MNP affected the child's current diet?

R: On the part of the diet, there is no observed change in the amount off food intake.

I: What about varieties of foods?

R: No. He doesn't select food.

I: OK. In other communities, we noticed that sharing of the MNP occurred. Could you talk any sharing of the MNP when you used it?

R: Honestly, it has never happen here. For those places where it occurs, I have never asked. I think it is given for a single child?

I: Yes

R: What is given for a single child, if the mother shared with others, it won't complete the sixty days. If at the end the product doesn't work, they don't have the right to say that it didn't work, since it is her fault for sharing. It is meant to be use for the number of days it is given. It is when it is given for that number of days that they can truly assess the effectiveness of the

MNP. IF the period of sixty days is over, the child supposed to be observed for any change. But if she shares it with another woman and it finish before the end of the sixty days, there will be problem there. The male caregivers need to help the mothers to avoid this.

I: Ok. Thank you for that explanation. Tell me about sharing of the MNP with other children in household or community

R: Honestly, it has never happened in my household.

I: Ok. What about you eating out of the MNP as a snack?

R: No. ... (Silence)

I: Ok. (Silence)

R: Yes. because whatever is for the child, it is supposed to be given to the child alone. Not to be taken by the parents.

I: OK. What about the consumption of the leftovers of the MNP?

R: After adding the MNP and he doesn't finish it, we cover it and give it to him later.

I: Ok. In your thinking, would the sixty sachets be enough for the sixty days?

R: Yes. By the grace of God. Since it is one per day for sixty days, it will be enough.

I: Why do you think it would be enough||||||?

R: |since it is one sachet per day and there are sixty sachets all together, they will be enough for the sixty days. One per day.

I: Now that you are using the MNP, could you talk about how you perceive it?

R: Honestly, what I will say is alhamdullilahi. Since we started giving him, even the regular fever he used to experience is not longer experienced. Yes. When i came back, I asked her and she told me that since she started giving the MNP, the child's health has not remain the same. I will only say I am grateful.

I: Thank you so much for the explanation. On the MNP itself, do you see it as food, medicine or as something else?

R: Honestly, I can say as medicine. ....

I: (Silence)

R: Because whatsoever you give to a person and it helps the body could be called medicine. Because, what will be food is what we just be eaten. If it is something that is taken without any improvement in one’s health, it can be referred to as food. Since it is improving child's health, it can be called medicine.

I: If this product is cheaply made available in the market in your community, would you purchase it?

R: Honestly, I have to enquire from the Health workers. Because, you may just see it and buy, which may end up been the wrong one or fake. It won't be good like that.

I: What of when you are sure of it?

R: I will buy......

I: what will make you to buy?

R: Because I have used and enjoyed it. I know the benefits of using it to the child. One can buy it.

I: How much would you be willing to pay for each sachet?

R: This sachet (pointing at a sachet of MNP)?

I: Yes.

R: Honestly, whatever is called a medicine, as musch as one knows the use, one cannot say it is too costly.

I: Like how much for this?

R: You see hundred naira? I can buy a sachet for it.

I: Thank you so much. We are making progress. You have been giving me great information. Now, please tell me some ways that you believe would be effective for promoting MNP to caregivers in the community so that they would comply with using it appropriately?

R: Honestly, if things like this come up, there should be advocacy on magaji, Dikko and Sarkin Fulani and explanation made to them. They will be the ones to go and explain to their fellow fulanis, there would be acceptance. But if someone just see you and he doesn't know you, and you bring things to him, even if you explain to him, he may not accept. But if he sees the leaders at the front campaigning, they will accept because they know and respect them. They will accept 100%.

I: Thank you. What ways would be effective to promote MNP for its appropriate use?

R: Honestly, this is a nice question. It can be said when was the medicine brought? How many days from today? Have you been using it appropriately? How many do you have left? Ok. Bring it. It should be counted and the remaining ones will give the idea whether the person is using or not. (Silence)

I: I am listening.

R: If there is imbalance in the calculation of the days and the number remaining, the person has not been using it appropriately. The person will need to be explained to the effect of not adhering strictly to the instruction. More awareness should be created on how it is used and made to know it is free, a help for their children health. So they should reciprocate by using it appropriately because the time will come when they will have to buy with their money and may not even be available in the market. IF they use it very well, it will help to prevent the children from becoming ill. When people see their neighbour's children who use it appropriately not falling ill often like theirs, they will ask. The one that didn't use it appropriately will also want to see her child becoming healthier. This will make her to start using it appropriately.

I: Thanks so much for that good suggestion. What people most influence your opinions and knowledge about feeding practices?

R: Honestly, the Health workers are the ones that can influence me on that. This is because they are the ones that know what to do and how to do it. Because even when they give you drugs, they will have to explain in details. So it is necessary that the health workers should be the ones to influence people's opinion.

I: Ok. Thank you. Who influences your decisions regarding feeding your baby the MNP?

R: The medical workers are the ones I got my influence from. They explain to us the benefit and all other things related to the MNP.

I: Thanks. Where would be good places to disseminate health information related to MNP?

R: These health workers are the ones I will always go to them for information. There are Hospitals here in Jega and in Aliero, I can go there to get information. Yes.

I: OK. What makes you think that way?

R: Because what you don't know and you are given, you need to go and ask to get information on the benefits. When you ask and the benefits explained to you, you will make good use of it and even encourage others to make use of it appropriately.

I: Who, in your community do you trust the most for receiving messages about infant and young child feeding?

R: I trust Ahmadu. Whatsoever he comes and tells me, I will believe.

I: Who is the Ahmadu?

R: He is the younger brother to Dikko.

I: OK. Thank you so much for the information. What forms of media do you prefer for receiving MNP Messages?

R: There is none like the one you're doing now, through word-of-mouth. I have been able to get a lot of explanation from you. If you leave and I still need more information, I will go and ask.

I: Ok. What about posters/brochure/flyers?

R: That one, my level of education has not got to that level.

I: You mentioned word-of-mouth, whom who you want to get such information from?

R: Through the health workers I told you earlier, one can go to them and get explanation. Like through the posters you mentioned, they can use it to explain to us.

I: Ok. What about radio?

R: If one listens to his radio, he will get detail information from it.

I: What about SMS?

R: Well, my level of education is not up to that level.

I: Is there any other media that you will suggest?

R: Ok. The form of media I prefer is like the one we just engaged in and even the health workers. For me, words-of-mouth are the best, since most of us cannot read or write.

I: What ways would you prefer of receiving education regarding IYCF and MNP?

R: We still have to go back to the health workers. If one goes to them, they will teach one a lot of things that one can use and even explain to others.

I: Ok. Tell me about specific examples of other successful health education campaigns in this community.

R: I think there are campaigns on dusa, lala like that. There were successful.

I: What made them successful?

R: Madalla. Because they come during heat period. At that time, a lot of children do die. When the vaccine was developed, it became successful because people who are vaccinated don't die. The vaccination made it successful.

I: What community channels will be the best to distribute the product?

R: Honestly, there is no better channel tyhan through the health workers. If it is just brought to the community like that, the people that even need it may end up not getting it. They may just dump it in one place. But if it is given to health workers, they use to follow from village-

to-village and distribute. If it is brought through the community leader, he can be asked to gather his people. Some people may find it difficult to travel to the hospital just to go and collect it since they don't know the importance. But since there are health workers who move from one village to another, people are using what they bring. That is the best channel to follow.

I: Thank you so much. I want to hear some specific messages you think would effectively promote the product.

R: Honestly, for the women, there is need for much wisdom. A female suppose to be clean, cleaning the child always, washing of clothes, covering of foods and other hygienic activities. For the man, when he comes back home, he should always remind the woman on the use of the MNP. If she has forgotten, the man should ask her to do that immediately. There is need for him to query her very well so that she won't forget again. Even if she forgets next time, she will quickly remember and give the child immediately at the sight of the husband from a distance. The man has to be involved in the programme to make it successful.

I: Ok. What specific messages should be used to ensure that it will be used appropriately?

R: There should be more enlightenment to the caregivers. They should know that the child will be healthy, eats well, strong and learn to walk quicker if he is given the product appropriately. If he walks faster, she will enjoy by sending him to do little things for her. If they know of these benefits, they will ensure they use it appropriately.

I: Thank you. What messages will be effective to promote its use but limit the sharing of it?

R: The man should always check and see what they are doing, showing interest to encourage her. The mother that is careless, the man should be strict on her to use it very well. But if it is brought and the man just give her without ever asking her about it, she may just go and dump it somewhere till it even expires. It won't be nice since it was brought to assist. If it is ones money he spent to buy, he will be more serious to use it well.

I: Finally, is there anything else that you can share about your experience with this product?

R: I will only recommend that health workers should be use to distribute this products around. That's all.

I: Ok. Thank you so much for your time.

R: Thanks for coming.
